# Supplementary material for: Modeling glioblastoma heterogeneity as a dynamic network of cell states
Source: Mol Syst Biol. 2021 Sep 16;17(9):e10105. doi: 10.15252/msb.202010105 (PMC8444284; doi:10.15252/msb.202010105)
Supplement: Supplementary file 5 — Source Data for Figure 3 [file MSB-17-e10105-s001.zip › Figure3A_sourcedata/GSEA_3065/nosigdb_state6.GseaPreranked.1622718443993/DOROSZKO_U3180MG_CLUSTER_2.html]

Details for gene set DOROSZKO\_U3180MG\_CLUSTER\_2[GSEA]

|  || Dataset | state6 |
| Phenotype | NoPhenotypeAvailable |
| Upregulated in class | na\_neg |
| GeneSet | DOROSZKO\_U3180MG\_CLUSTER\_2 |
| Enrichment Score (ES) | -0.60710496 |
| Normalized Enrichment Score (NES) | -2.9035587 |
| Nominal p-value | 0.0 |
| FDR q-value | 0.0 |
| FWER p-Value | 0.0 |
Table: GSEA Results Summary

  

Fig 1: Enrichment plot: DOROSZKO\_U3180MG\_CLUSTER\_2      
 Profile of the Running ES Score & Positions of GeneSet Members on the Rank Ordered List

  

| PROBE | GENE SYMBOL | GENE\_TITLE | RANK IN GENE LIST | RANK METRIC SCORE | RUNNING ES | CORE ENRICHMENT || 1 | GADD45A |  |  | 2 | 0.419 | 0.0093 | No |
| 2 | DLL3 |  |  | 49 | 0.246 | 0.0094 | No |
| 3 | PPP1CB |  |  | 143 | 0.196 | 0.0026 | No |
| 4 | DDIT3 |  |  | 205 | 0.175 | -0.0007 | No |
| 5 | GTF2I |  |  | 284 | 0.154 | -0.0066 | No |
| 6 | HNRNPH1 |  |  | 378 | 0.139 | -0.0146 | No |
| 7 | YBX3 |  |  | 432 | 0.131 | -0.0180 | No |
| 8 | NIN |  |  | 542 | 0.119 | -0.0283 | No |
| 9 | PRRC2B |  |  | 711 | 0.105 | -0.0461 | No |
| 10 | SAT1 |  |  | 744 | 0.102 | -0.0476 | No |
| 11 | HSPA1B |  |  | 814 | 0.097 | -0.0537 | No |
| 12 | ATRX |  |  | 861 | 0.094 | -0.0571 | No |
| 13 | IP6K2 |  |  | 1001 | 0.086 | -0.0718 | No |
| 14 | SRSF11 |  |  | 1095 | 0.081 | -0.0811 | No |
| 15 | FAM120A |  |  | 1110 | 0.080 | -0.0810 | No |
| 16 | TIA1 |  |  | 1177 | 0.076 | -0.0872 | No |
| 17 | BAZ2B |  |  | 1208 | 0.075 | -0.0891 | No |
| 18 | CREB5 |  |  | 1231 | 0.074 | -0.0900 | No |
| 19 | TCF4 |  |  | 1234 | 0.074 | -0.0886 | No |
| 20 | KMT2A |  |  | 1276 | 0.072 | -0.0919 | No |
| 21 | CLK1 |  |  | 1366 | 0.069 | -0.1010 | No |
| 22 | TBL1XR1 |  |  | 1386 | 0.068 | -0.1017 | No |
| 23 | ACAP3 |  |  | 1434 | 0.067 | -0.1058 | No |
| 24 | KMT5B |  |  | 1452 | 0.066 | -0.1064 | No |
| 25 | CTNNB1 |  |  | 1461 | 0.066 | -0.1058 | No |
| 26 | MTA1 |  |  | 1542 | 0.062 | -0.1140 | No |
| 27 | SAFB2 |  |  | 1544 | 0.062 | -0.1127 | No |
| 28 | ARRDC3 |  |  | 1579 | 0.061 | -0.1154 | No |
| 29 | LIMA1 |  |  | 1702 | 0.057 | -0.1288 | No |
| 30 | USP34 |  |  | 1740 | 0.055 | -0.1320 | No |
| 31 | ARIH2 |  |  | 1767 | 0.055 | -0.1338 | No |
| 32 | PHF20 |  |  | 1801 | 0.054 | -0.1366 | No |
| 33 | SERINC1 |  |  | 1811 | 0.053 | -0.1364 | No |
| 34 | ANAPC5 |  |  | 1859 | 0.052 | -0.1409 | No |
| 35 | ZBTB20 |  |  | 1892 | 0.051 | -0.1436 | No |
| 36 | RAF1 |  |  | 1924 | 0.050 | -0.1462 | No |
| 37 | TIMM44 |  |  | 1930 | 0.050 | -0.1456 | No |
| 38 | ACADVL |  |  | 1940 | 0.049 | -0.1456 | No |
| 39 | SNHG9 |  |  | 2069 | 0.046 | -0.1599 | No |
| 40 | PPP1R16A |  |  | 2075 | 0.046 | -0.1595 | No |
| 41 | MYC |  |  | 2081 | 0.045 | -0.1591 | No |
| 42 | IRS2 |  |  | 2158 | 0.043 | -0.1672 | No |
| 43 | FOXO3 |  |  | 2178 | 0.042 | -0.1685 | No |
| 44 | THAP5 |  |  | 2192 | 0.042 | -0.1691 | No |
| 45 | PTCD3 |  |  | 2224 | 0.040 | -0.1719 | No |
| 46 | JAG1 |  |  | 2234 | 0.040 | -0.1721 | No |
| 47 | RFX4 |  |  | 2238 | 0.040 | -0.1715 | No |
| 48 | TLK1 |  |  | 2244 | 0.040 | -0.1712 | No |
| 49 | CDC42BPA |  |  | 2279 | 0.039 | -0.1744 | No |
| 50 | MTRF1L |  |  | 2280 | 0.039 | -0.1735 | No |
| 51 | ABI2 |  |  | 2307 | 0.038 | -0.1758 | No |
| 52 | DHX30 |  |  | 2309 | 0.038 | -0.1750 | No |
| 53 | MED6 |  |  | 2312 | 0.038 | -0.1744 | No |
| 54 | NCSTN |  |  | 2327 | 0.038 | -0.1752 | No |
| 55 | CHD1 |  |  | 2339 | 0.038 | -0.1757 | No |
| 56 | TRIM44 |  |  | 2341 | 0.038 | -0.1749 | No |
| 57 | SETD5 |  |  | 2458 | 0.035 | -0.1881 | No |
| 58 | DDX6 |  |  | 2557 | 0.032 | -0.1991 | No |
| 59 | FERMT1 |  |  | 2598 | 0.031 | -0.2032 | No |
| 60 | DAAM1 |  |  | 2604 | 0.031 | -0.2031 | No |
| 61 | ZNF131 |  |  | 2610 | 0.031 | -0.2030 | No |
| 62 | PUS7L |  |  | 2671 | 0.029 | -0.2095 | No |
| 63 | JMJD1C |  |  | 2733 | 0.028 | -0.2162 | No |
| 64 | DDX18 |  |  | 2755 | 0.028 | -0.2181 | No |
| 65 | ARHGEF7 |  |  | 2777 | 0.027 | -0.2200 | No |
| 66 | SEC61A1 |  |  | 2806 | 0.026 | -0.2228 | No |
| 67 | TMEM120A |  |  | 2820 | 0.026 | -0.2237 | No |
| 68 | PLPP3 |  |  | 2894 | 0.024 | -0.2320 | No |
| 69 | GCC2 |  |  | 2910 | 0.024 | -0.2332 | No |
| 70 | RAB3IP |  |  | 2911 | 0.024 | -0.2327 | No |
| 71 | RPAP2 |  |  | 2915 | 0.024 | -0.2325 | No |
| 72 | HSPA4 |  |  | 2968 | 0.023 | -0.2382 | No |
| 73 | KMT2E |  |  | 2983 | 0.022 | -0.2394 | No |
| 74 | DCLK2 |  |  | 3031 | 0.021 | -0.2445 | No |
| 75 | FOXN2 |  |  | 3044 | 0.021 | -0.2455 | No |
| 76 | RB1CC1 |  |  | 3107 | 0.020 | -0.2525 | No |
| 77 | SRSF6 |  |  | 3114 | 0.020 | -0.2528 | No |
| 78 | PKN2 |  |  | 3131 | 0.019 | -0.2543 | No |
| 79 | ESF1 |  |  | 3204 | 0.018 | -0.2625 | No |
| 80 | CPEB4 |  |  | 3220 | 0.017 | -0.2639 | No |
| 81 | MPZL1 |  |  | 3226 | 0.017 | -0.2641 | No |
| 82 | TAOK1 |  |  | 3341 | 0.015 | -0.2775 | No |
| 83 | ABCE1 |  |  | 3395 | 0.013 | -0.2835 | No |
| 84 | PSRC1 |  |  | 3406 | 0.013 | -0.2844 | No |
| 85 | TRA2A |  |  | 3410 | 0.013 | -0.2845 | No |
| 86 | MCL1 |  |  | 3430 | 0.012 | -0.2865 | No |
| 87 | TNRC6B |  |  | 3434 | 0.012 | -0.2866 | No |
| 88 | CFLAR |  |  | 3461 | 0.012 | -0.2894 | No |
| 89 | CCDC82 |  |  | 3488 | 0.011 | -0.2923 | No |
| 90 | CRTC3 |  |  | 3505 | 0.011 | -0.2940 | No |
| 91 | PHF14 |  |  | 3551 | 0.010 | -0.2991 | No |
| 92 | TMUB2 |  |  | 3561 | 0.010 | -0.3000 | No |
| 93 | ANK2 |  |  | 3576 | 0.010 | -0.3014 | No |
| 94 | NCOR1 |  |  | 3732 | 0.006 | -0.3199 | No |
| 95 | AFF4 |  |  | 3845 | 0.004 | -0.3333 | No |
| 96 | EPN2 |  |  | 3885 | 0.003 | -0.3379 | No |
| 97 | ZNF37A |  |  | 3902 | 0.002 | -0.3397 | No |
| 98 | NSD1 |  |  | 3919 | 0.002 | -0.3416 | No |
| 99 | DMAP1 |  |  | 3920 | 0.002 | -0.3416 | No |
| 100 | DHX36 |  |  | 3924 | 0.002 | -0.3419 | No |
| 101 | SAT2 |  |  | 4051 | -0.000 | -0.3570 | No |
| 102 | NPAS3 |  |  | 4053 | -0.000 | -0.3571 | No |
| 103 | DDB1 |  |  | 4082 | -0.001 | -0.3605 | No |
| 104 | TAOK3 |  |  | 4090 | -0.001 | -0.3613 | No |
| 105 | SPTBN1 |  |  | 4188 | -0.003 | -0.3728 | No |
| 106 | RAB6A |  |  | 4227 | -0.004 | -0.3773 | No |
| 107 | FAM53C |  |  | 4234 | -0.004 | -0.3779 | No |
| 108 | ATXN7L3B |  |  | 4235 | -0.004 | -0.3779 | No |
| 109 | SYT11 |  |  | 4256 | -0.004 | -0.3802 | No |
| 110 | DTNA |  |  | 4259 | -0.004 | -0.3803 | No |
| 111 | CHD9 |  |  | 4316 | -0.005 | -0.3869 | No |
| 112 | ASH1L |  |  | 4327 | -0.005 | -0.3880 | No |
| 113 | XBP1 |  |  | 4331 | -0.006 | -0.3882 | No |
| 114 | MYCBP2 |  |  | 4340 | -0.006 | -0.3890 | No |
| 115 | KIF1B |  |  | 4348 | -0.006 | -0.3898 | No |
| 116 | ADGRG1 |  |  | 4417 | -0.007 | -0.3978 | No |
| 117 | SLC35F1 |  |  | 4421 | -0.007 | -0.3979 | No |
| 118 | VPS37B |  |  | 4441 | -0.008 | -0.4001 | No |
| 119 | CSNK2A1 |  |  | 4449 | -0.008 | -0.4007 | No |
| 120 | MLF1 |  |  | 4462 | -0.008 | -0.4020 | No |
| 121 | CNTN1 |  |  | 4467 | -0.008 | -0.4023 | No |
| 122 | SLC25A36 |  |  | 4534 | -0.009 | -0.4100 | No |
| 123 | RBM26 |  |  | 4542 | -0.010 | -0.4106 | No |
| 124 | GNPAT |  |  | 4548 | -0.010 | -0.4110 | No |
| 125 | DTX3 |  |  | 4633 | -0.011 | -0.4208 | No |
| 126 | SEMA5A |  |  | 4642 | -0.012 | -0.4215 | No |
| 127 | MARK3 |  |  | 4659 | -0.012 | -0.4231 | No |
| 128 | MEIS2 |  |  | 4714 | -0.013 | -0.4293 | No |
| 129 | ITGAV |  |  | 4722 | -0.013 | -0.4299 | No |
| 130 | SMCHD1 |  |  | 4732 | -0.013 | -0.4307 | No |
| 131 | ZEB2 |  |  | 4735 | -0.013 | -0.4306 | No |
| 132 | WDR60 |  |  | 4750 | -0.013 | -0.4320 | No |
| 133 | SDC3 |  |  | 4777 | -0.014 | -0.4348 | No |
| 134 | AHCYL1 |  |  | 4786 | -0.014 | -0.4354 | No |
| 135 | TERF1 |  |  | 4804 | -0.014 | -0.4371 | No |
| 136 | TUFM |  |  | 4837 | -0.015 | -0.4406 | No |
| 137 | IDS |  |  | 4847 | -0.015 | -0.4414 | No |
| 138 | GDAP1 |  |  | 4904 | -0.017 | -0.4477 | No |
| 139 | GNL3 |  |  | 4977 | -0.018 | -0.4559 | No |
| 140 | GRN |  |  | 4995 | -0.018 | -0.4576 | No |
| 141 | STAT3 |  |  | 5004 | -0.018 | -0.4581 | No |
| 142 | LRP10 |  |  | 5011 | -0.018 | -0.4584 | No |
| 143 | RNF170 |  |  | 5061 | -0.020 | -0.4638 | No |
| 144 | COLGALT2 |  |  | 5075 | -0.020 | -0.4649 | No |
| 145 | RBM4B |  |  | 5095 | -0.020 | -0.4668 | No |
| 146 | ADAR |  |  | 5098 | -0.020 | -0.4665 | No |
| 147 | SPTAN1 |  |  | 5102 | -0.020 | -0.4664 | No |
| 148 | REV3L |  |  | 5120 | -0.021 | -0.4680 | No |
| 149 | CNOT2 |  |  | 5208 | -0.022 | -0.4779 | No |
| 150 | SACS |  |  | 5232 | -0.023 | -0.4802 | No |
| 151 | HAGHL |  |  | 5237 | -0.023 | -0.4801 | No |
| 152 | LAMP1 |  |  | 5265 | -0.024 | -0.4828 | No |
| 153 | NELFCD |  |  | 5271 | -0.024 | -0.4829 | No |
| 154 | RDH11 |  |  | 5274 | -0.024 | -0.4826 | No |
| 155 | RSBN1L |  |  | 5292 | -0.024 | -0.4841 | No |
| 156 | FKBP10 |  |  | 5319 | -0.025 | -0.4867 | No |
| 157 | EXOC4 |  |  | 5354 | -0.025 | -0.4902 | No |
| 158 | ARID4A |  |  | 5383 | -0.026 | -0.4929 | No |
| 159 | NCK1 |  |  | 5409 | -0.027 | -0.4953 | No |
| 160 | TLE4 |  |  | 5414 | -0.027 | -0.4952 | No |
| 161 | TMEM59L |  |  | 5431 | -0.027 | -0.4965 | No |
| 162 | ARHGEF2 |  |  | 5504 | -0.029 | -0.5045 | No |
| 163 | GPBP1 |  |  | 5513 | -0.029 | -0.5048 | No |
| 164 | TRMT13 |  |  | 5569 | -0.030 | -0.5107 | No |
| 165 | ZNF655 |  |  | 5572 | -0.030 | -0.5103 | No |
| 166 | MAGI1 |  |  | 5589 | -0.030 | -0.5115 | No |
| 167 | SPSB3 |  |  | 5607 | -0.031 | -0.5129 | No |
| 168 | FNIP1 |  |  | 5622 | -0.031 | -0.5138 | No |
| 169 | MYO6 |  |  | 5677 | -0.032 | -0.5196 | No |
| 170 | EXOC7 |  |  | 5690 | -0.032 | -0.5203 | No |
| 171 | RPRD1A |  |  | 5704 | -0.032 | -0.5211 | No |
| 172 | ATP6V0B |  |  | 5714 | -0.033 | -0.5215 | No |
| 173 | IAH1 |  |  | 5727 | -0.033 | -0.5221 | No |
| 174 | SRSF5 |  |  | 5739 | -0.033 | -0.5227 | No |
| 175 | FASTK |  |  | 5745 | -0.033 | -0.5225 | No |
| 176 | DDX42 |  |  | 5835 | -0.035 | -0.5324 | No |
| 177 | RIMKLB |  |  | 5883 | -0.036 | -0.5373 | No |
| 178 | DNPEP |  |  | 5907 | -0.036 | -0.5392 | No |
| 179 | PODXL2 |  |  | 5938 | -0.037 | -0.5420 | No |
| 180 | RIF1 |  |  | 5945 | -0.037 | -0.5418 | No |
| 181 | CEBPZ |  |  | 5976 | -0.038 | -0.5446 | No |
| 182 | TUBGCP2 |  |  | 5982 | -0.038 | -0.5443 | No |
| 183 | CAMK2D |  |  | 6020 | -0.039 | -0.5479 | No |
| 184 | GPRC5B |  |  | 6049 | -0.039 | -0.5504 | No |
| 185 | NAV1 |  |  | 6064 | -0.039 | -0.5512 | No |
| 186 | YME1L1 |  |  | 6080 | -0.040 | -0.5520 | No |
| 187 | CCDC88A |  |  | 6086 | -0.040 | -0.5517 | No |
| 188 | CEBPZOS |  |  | 6091 | -0.040 | -0.5513 | No |
| 189 | SECISBP2 |  |  | 6106 | -0.040 | -0.5521 | No |
| 190 | TTC37 |  |  | 6163 | -0.042 | -0.5578 | No |
| 191 | PBXIP1 |  |  | 6181 | -0.042 | -0.5589 | No |
| 192 | ORAI2 |  |  | 6195 | -0.043 | -0.5595 | No |
| 193 | GOLGB1 |  |  | 6206 | -0.043 | -0.5597 | No |
| 194 | BCLAF1 |  |  | 6210 | -0.043 | -0.5591 | No |
| 195 | GPATCH2 |  |  | 6213 | -0.043 | -0.5584 | No |
| 196 | SRSF2 |  |  | 6250 | -0.044 | -0.5617 | No |
| 197 | RIC3 |  |  | 6255 | -0.044 | -0.5612 | No |
| 198 | CYHR1 |  |  | 6272 | -0.044 | -0.5621 | No |
| 199 | USP33 |  |  | 6299 | -0.044 | -0.5642 | No |
| 200 | ZNF638 |  |  | 6302 | -0.045 | -0.5635 | No |
| 201 | TRA2B |  |  | 6303 | -0.045 | -0.5625 | No |
| 202 | HECTD1 |  |  | 6311 | -0.045 | -0.5623 | No |
| 203 | TCERG1 |  |  | 6317 | -0.045 | -0.5619 | No |
| 204 | COPA |  |  | 6322 | -0.045 | -0.5613 | No |
| 205 | CELF1 |  |  | 6338 | -0.045 | -0.5621 | No |
| 206 | NCAM1 |  |  | 6342 | -0.046 | -0.5614 | No |
| 207 | USP3 |  |  | 6344 | -0.046 | -0.5605 | No |
| 208 | TAF1D |  |  | 6372 | -0.047 | -0.5627 | No |
| 209 | EMD |  |  | 6376 | -0.047 | -0.5620 | No |
| 210 | HOOK3 |  |  | 6406 | -0.047 | -0.5644 | No |
| 211 | AUP1 |  |  | 6407 | -0.047 | -0.5633 | No |
| 212 | MKLN1 |  |  | 6410 | -0.047 | -0.5625 | No |
| 213 | UBR5 |  |  | 6426 | -0.048 | -0.5632 | No |
| 214 | HEPACAM |  |  | 6448 | -0.049 | -0.5646 | No |
| 215 | CCDC66 |  |  | 6507 | -0.050 | -0.5704 | No |
| 216 | PSMC6 |  |  | 6525 | -0.050 | -0.5713 | No |
| 217 | SRSF10 |  |  | 6530 | -0.050 | -0.5707 | No |
| 218 | GPATCH8 |  |  | 6532 | -0.051 | -0.5696 | No |
| 219 | ELMSAN1 |  |  | 6549 | -0.051 | -0.5704 | No |
| 220 | SRSF7 |  |  | 6593 | -0.053 | -0.5743 | No |
| 221 | PRRC2A |  |  | 6599 | -0.053 | -0.5737 | No |
| 222 | FAM172A |  |  | 6605 | -0.053 | -0.5731 | No |
| 223 | HSPH1 |  |  | 6617 | -0.053 | -0.5732 | No |
| 224 | SRSF1 |  |  | 6655 | -0.054 | -0.5765 | No |
| 225 | HERPUD1 |  |  | 6698 | -0.056 | -0.5802 | No |
| 226 | PSAP |  |  | 6739 | -0.057 | -0.5837 | No |
| 227 | QTRT1 |  |  | 6751 | -0.057 | -0.5837 | No |
| 228 | ABHD5 |  |  | 6764 | -0.058 | -0.5839 | No |
| 229 | KMT2C |  |  | 6775 | -0.058 | -0.5837 | No |
| 230 | OSBPL1A |  |  | 6789 | -0.058 | -0.5840 | No |
| 231 | RBM39 |  |  | 6825 | -0.059 | -0.5868 | No |
| 232 | RBM28 |  |  | 6834 | -0.060 | -0.5864 | No |
| 233 | DPP6 |  |  | 6845 | -0.060 | -0.5863 | No |
| 234 | CDK5RAP3 |  |  | 6851 | -0.060 | -0.5855 | No |
| 235 | CDC37 |  |  | 6861 | -0.061 | -0.5852 | No |
| 236 | ANAPC7 |  |  | 6890 | -0.062 | -0.5872 | No |
| 237 | PTK2 |  |  | 6894 | -0.062 | -0.5861 | No |
| 238 | SPAG9 |  |  | 6926 | -0.062 | -0.5884 | No |
| 239 | M6PR |  |  | 6966 | -0.064 | -0.5916 | No |
| 240 | SLC22A17 |  |  | 6968 | -0.064 | -0.5903 | No |
| 241 | RBM23 |  |  | 7025 | -0.066 | -0.5955 | No |
| 242 | COMMD2 |  |  | 7062 | -0.067 | -0.5983 | No |
| 243 | HNRNPU |  |  | 7074 | -0.067 | -0.5981 | No |
| 244 | CCNL1 |  |  | 7107 | -0.068 | -0.6004 | No |
| 245 | SLC3A2 |  |  | 7118 | -0.068 | -0.6001 | No |
| 246 | IST1 |  |  | 7142 | -0.069 | -0.6013 | No |
| 247 | CTSD |  |  | 7147 | -0.069 | -0.6002 | No |
| 248 | DPF3 |  |  | 7149 | -0.069 | -0.5987 | No |
| 249 | IFRD1 |  |  | 7168 | -0.070 | -0.5993 | No |
| 250 | MPHOSPH8 |  |  | 7195 | -0.071 | -0.6008 | No |
| 251 | PRPF4B |  |  | 7247 | -0.073 | -0.6053 | No |
| 252 | OFD1 |  |  | 7259 | -0.073 | -0.6049 | No |
| 253 | B3GAT3 |  |  | 7267 | -0.073 | -0.6041 | No |
| 254 | PTBP1 |  |  | 7293 | -0.074 | -0.6054 | Yes |
| 255 | ZNF800 |  |  | 7296 | -0.074 | -0.6040 | Yes |
| 256 | HES1 |  |  | 7301 | -0.074 | -0.6028 | Yes |
| 257 | CTSF |  |  | 7309 | -0.075 | -0.6019 | Yes |
| 258 | CHD6 |  |  | 7317 | -0.075 | -0.6010 | Yes |
| 259 | CWC25 |  |  | 7322 | -0.075 | -0.5998 | Yes |
| 260 | SNRNP70 |  |  | 7343 | -0.076 | -0.6005 | Yes |
| 261 | SMARCC2 |  |  | 7361 | -0.077 | -0.6008 | Yes |
| 262 | KNOP1 |  |  | 7387 | -0.077 | -0.6020 | Yes |
| 263 | KIF21A |  |  | 7417 | -0.078 | -0.6037 | Yes |
| 264 | IFRD2 |  |  | 7423 | -0.078 | -0.6026 | Yes |
| 265 | HSPD1 |  |  | 7433 | -0.079 | -0.6018 | Yes |
| 266 | AKAP9 |  |  | 7436 | -0.079 | -0.6003 | Yes |
| 267 | N4BP2L2 |  |  | 7458 | -0.080 | -0.6010 | Yes |
| 268 | SLC12A9 |  |  | 7472 | -0.080 | -0.6007 | Yes |
| 269 | PRCP |  |  | 7495 | -0.081 | -0.6015 | Yes |
| 270 | NOVA1 |  |  | 7506 | -0.082 | -0.6009 | Yes |
| 271 | U2SURP |  |  | 7509 | -0.082 | -0.5993 | Yes |
| 272 | ABHD12 |  |  | 7521 | -0.082 | -0.5987 | Yes |
| 273 | PRPSAP1 |  |  | 7540 | -0.083 | -0.5990 | Yes |
| 274 | MAT2A |  |  | 7544 | -0.084 | -0.5974 | Yes |
| 275 | ZNF302 |  |  | 7546 | -0.084 | -0.5957 | Yes |
| 276 | AC020916.1 |  |  | 7554 | -0.084 | -0.5946 | Yes |
| 277 | ARHGAP21 |  |  | 7574 | -0.085 | -0.5949 | Yes |
| 278 | LFNG |  |  | 7578 | -0.085 | -0.5934 | Yes |
| 279 | SYNE2 |  |  | 7598 | -0.086 | -0.5937 | Yes |
| 280 | TDG |  |  | 7606 | -0.086 | -0.5926 | Yes |
| 281 | SNRNP200 |  |  | 7608 | -0.086 | -0.5907 | Yes |
| 282 | PJA2 |  |  | 7613 | -0.087 | -0.5892 | Yes |
| 283 | DPF2 |  |  | 7623 | -0.087 | -0.5883 | Yes |
| 284 | HEPN1 |  |  | 7644 | -0.088 | -0.5887 | Yes |
| 285 | SF3B1 |  |  | 7654 | -0.089 | -0.5878 | Yes |
| 286 | SLC39A1 |  |  | 7656 | -0.089 | -0.5859 | Yes |
| 287 | SFPQ |  |  | 7661 | -0.089 | -0.5844 | Yes |
| 288 | ARFGAP2 |  |  | 7672 | -0.089 | -0.5836 | Yes |
| 289 | SUPT20H |  |  | 7691 | -0.091 | -0.5837 | Yes |
| 290 | THOC2 |  |  | 7693 | -0.091 | -0.5817 | Yes |
| 291 | SON |  |  | 7704 | -0.091 | -0.5808 | Yes |
| 292 | NEMF |  |  | 7735 | -0.093 | -0.5823 | Yes |
| 293 | LRP1 |  |  | 7748 | -0.093 | -0.5817 | Yes |
| 294 | CTSA |  |  | 7751 | -0.093 | -0.5798 | Yes |
| 295 | FAM133B |  |  | 7753 | -0.093 | -0.5778 | Yes |
| 296 | PBRM1 |  |  | 7765 | -0.094 | -0.5770 | Yes |
| 297 | EIF2S3 |  |  | 7783 | -0.094 | -0.5769 | Yes |
| 298 | TNFRSF12A |  |  | 7784 | -0.094 | -0.5747 | Yes |
| 299 | TCF25 |  |  | 7795 | -0.095 | -0.5738 | Yes |
| 300 | GLG1 |  |  | 7811 | -0.096 | -0.5734 | Yes |
| 301 | TMEM30A |  |  | 7814 | -0.096 | -0.5714 | Yes |
| 302 | GTF2H2 |  |  | 7815 | -0.097 | -0.5692 | Yes |
| 303 | MUM1 |  |  | 7817 | -0.097 | -0.5671 | Yes |
| 304 | MACF1 |  |  | 7819 | -0.097 | -0.5651 | Yes |
| 305 | JPX |  |  | 7824 | -0.097 | -0.5633 | Yes |
| 306 | DDR1 |  |  | 7836 | -0.098 | -0.5624 | Yes |
| 307 | NSUN5 |  |  | 7837 | -0.098 | -0.5602 | Yes |
| 308 | BDP1 |  |  | 7840 | -0.098 | -0.5582 | Yes |
| 309 | OS9 |  |  | 7859 | -0.099 | -0.5581 | Yes |
| 310 | GPM6A |  |  | 7875 | -0.100 | -0.5577 | Yes |
| 311 | ATF6B |  |  | 7888 | -0.101 | -0.5568 | Yes |
| 312 | IFNGR1 |  |  | 7892 | -0.101 | -0.5549 | Yes |
| 313 | PGP |  |  | 7898 | -0.101 | -0.5532 | Yes |
| 314 | NOC2L |  |  | 7926 | -0.102 | -0.5541 | Yes |
| 315 | CHMP4A |  |  | 7946 | -0.104 | -0.5540 | Yes |
| 316 | DNAJC2 |  |  | 7949 | -0.104 | -0.5519 | Yes |
| 317 | SPPL2A |  |  | 7951 | -0.104 | -0.5497 | Yes |
| 318 | DDX46 |  |  | 7980 | -0.106 | -0.5506 | Yes |
| 319 | MATN2 |  |  | 7984 | -0.106 | -0.5486 | Yes |
| 320 | ATP6AP1 |  |  | 7998 | -0.107 | -0.5477 | Yes |
| 321 | LONP2 |  |  | 8041 | -0.110 | -0.5502 | Yes |
| 322 | WEE1 |  |  | 8054 | -0.111 | -0.5492 | Yes |
| 323 | EPM2AIP1 |  |  | 8055 | -0.111 | -0.5466 | Yes |
| 324 | ZNF292 |  |  | 8071 | -0.111 | -0.5459 | Yes |
| 325 | CTTN |  |  | 8073 | -0.112 | -0.5435 | Yes |
| 326 | FGFR1 |  |  | 8080 | -0.112 | -0.5417 | Yes |
| 327 | DDX3X |  |  | 8095 | -0.113 | -0.5408 | Yes |
| 328 | SPARCL1 |  |  | 8104 | -0.114 | -0.5391 | Yes |
| 329 | FJX1 |  |  | 8110 | -0.115 | -0.5371 | Yes |
| 330 | RNF115 |  |  | 8111 | -0.115 | -0.5345 | Yes |
| 331 | MSI2 |  |  | 8115 | -0.115 | -0.5323 | Yes |
| 332 | LARS |  |  | 8123 | -0.116 | -0.5305 | Yes |
| 333 | GPAA1 |  |  | 8125 | -0.116 | -0.5280 | Yes |
| 334 | RTN4 |  |  | 8129 | -0.116 | -0.5257 | Yes |
| 335 | GOLIM4 |  |  | 8149 | -0.117 | -0.5253 | Yes |
| 336 | GATAD1 |  |  | 8159 | -0.118 | -0.5237 | Yes |
| 337 | CYR61 |  |  | 8178 | -0.120 | -0.5231 | Yes |
| 338 | NPDC1 |  |  | 8185 | -0.120 | -0.5211 | Yes |
| 339 | USP16 |  |  | 8187 | -0.121 | -0.5185 | Yes |
| 340 | ITGB8 |  |  | 8195 | -0.121 | -0.5166 | Yes |
| 341 | STX16 |  |  | 8220 | -0.123 | -0.5167 | Yes |
| 342 | LMNA |  |  | 8225 | -0.124 | -0.5143 | Yes |
| 343 | AIG1 |  |  | 8233 | -0.124 | -0.5124 | Yes |
| 344 | TMEM87A |  |  | 8247 | -0.126 | -0.5111 | Yes |
| 345 | CTSL |  |  | 8250 | -0.126 | -0.5084 | Yes |
| 346 | SELENOS |  |  | 8258 | -0.127 | -0.5064 | Yes |
| 347 | UNC50 |  |  | 8266 | -0.128 | -0.5043 | Yes |
| 348 | ST5 |  |  | 8269 | -0.128 | -0.5017 | Yes |
| 349 | WDR73 |  |  | 8270 | -0.129 | -0.4987 | Yes |
| 350 | LUC7L |  |  | 8271 | -0.129 | -0.4958 | Yes |
| 351 | ZNF680 |  |  | 8274 | -0.129 | -0.4931 | Yes |
| 352 | RNMT |  |  | 8277 | -0.129 | -0.4904 | Yes |
| 353 | NAA15 |  |  | 8290 | -0.131 | -0.4889 | Yes |
| 354 | CCS |  |  | 8294 | -0.132 | -0.4863 | Yes |
| 355 | ACSL3 |  |  | 8295 | -0.132 | -0.4833 | Yes |
| 356 | CCDC14 |  |  | 8299 | -0.132 | -0.4806 | Yes |
| 357 | NASP |  |  | 8318 | -0.134 | -0.4797 | Yes |
| 358 | PTX3 |  |  | 8319 | -0.134 | -0.4767 | Yes |
| 359 | MTF2 |  |  | 8320 | -0.134 | -0.4736 | Yes |
| 360 | DDRGK1 |  |  | 8324 | -0.135 | -0.4709 | Yes |
| 361 | CNOT4 |  |  | 8325 | -0.135 | -0.4679 | Yes |
| 362 | DNM1L |  |  | 8350 | -0.139 | -0.4676 | Yes |
| 363 | TTC19 |  |  | 8352 | -0.139 | -0.4646 | Yes |
| 364 | TM9SF2 |  |  | 8353 | -0.139 | -0.4614 | Yes |
| 365 | EFHC1 |  |  | 8356 | -0.140 | -0.4584 | Yes |
| 366 | B4GAT1 |  |  | 8363 | -0.141 | -0.4560 | Yes |
| 367 | WSB1 |  |  | 8366 | -0.142 | -0.4530 | Yes |
| 368 | CD164 |  |  | 8369 | -0.142 | -0.4500 | Yes |
| 369 | FSTL1 |  |  | 8373 | -0.143 | -0.4471 | Yes |
| 370 | CLU |  |  | 8387 | -0.145 | -0.4453 | Yes |
| 371 | ANKRD12 |  |  | 8389 | -0.145 | -0.4422 | Yes |
| 372 | CAND1 |  |  | 8405 | -0.147 | -0.4406 | Yes |
| 373 | PRPF38B |  |  | 8412 | -0.148 | -0.4380 | Yes |
| 374 | SLC16A1 |  |  | 8415 | -0.148 | -0.4349 | Yes |
| 375 | ZRANB2 |  |  | 8418 | -0.148 | -0.4317 | Yes |
| 376 | LAMP2 |  |  | 8425 | -0.149 | -0.4291 | Yes |
| 377 | MFGE8 |  |  | 8433 | -0.150 | -0.4265 | Yes |
| 378 | PRKCSH |  |  | 8441 | -0.151 | -0.4239 | Yes |
| 379 | BSG |  |  | 8443 | -0.151 | -0.4206 | Yes |
| 380 | RCN2 |  |  | 8451 | -0.152 | -0.4180 | Yes |
| 381 | BICD1 |  |  | 8452 | -0.153 | -0.4145 | Yes |
| 382 | HM13 |  |  | 8459 | -0.154 | -0.4117 | Yes |
| 383 | SAR1B |  |  | 8466 | -0.154 | -0.4089 | Yes |
| 384 | SPECC1 |  |  | 8467 | -0.154 | -0.4054 | Yes |
| 385 | PRKCA |  |  | 8485 | -0.158 | -0.4039 | Yes |
| 386 | ARHGAP5 |  |  | 8494 | -0.160 | -0.4012 | Yes |
| 387 | PCM1 |  |  | 8496 | -0.160 | -0.3977 | Yes |
| 388 | LRPAP1 |  |  | 8498 | -0.161 | -0.3941 | Yes |
| 389 | SPTLC1 |  |  | 8500 | -0.161 | -0.3906 | Yes |
| 390 | GON4L |  |  | 8509 | -0.163 | -0.3879 | Yes |
| 391 | PNN |  |  | 8524 | -0.167 | -0.3857 | Yes |
| 392 | P4HTM |  |  | 8529 | -0.168 | -0.3824 | Yes |
| 393 | SFXN5 |  |  | 8537 | -0.170 | -0.3794 | Yes |
| 394 | PABPN1 |  |  | 8553 | -0.173 | -0.3772 | Yes |
| 395 | GUSB |  |  | 8561 | -0.176 | -0.3741 | Yes |
| 396 | CLSTN1 |  |  | 8562 | -0.176 | -0.3701 | Yes |
| 397 | RGS12 |  |  | 8563 | -0.176 | -0.3661 | Yes |
| 398 | SF1 |  |  | 8565 | -0.177 | -0.3622 | Yes |
| 399 | ASPH |  |  | 8566 | -0.177 | -0.3582 | Yes |
| 400 | DDX5 |  |  | 8567 | -0.177 | -0.3542 | Yes |
| 401 | HMGXB4 |  |  | 8570 | -0.178 | -0.3504 | Yes |
| 402 | SUMF2 |  |  | 8583 | -0.184 | -0.3476 | Yes |
| 403 | RCN1 |  |  | 8602 | -0.191 | -0.3455 | Yes |
| 404 | TMEM165 |  |  | 8605 | -0.194 | -0.3413 | Yes |
| 405 | RSRP1 |  |  | 8606 | -0.195 | -0.3369 | Yes |
| 406 | ATP6AP2 |  |  | 8607 | -0.195 | -0.3324 | Yes |
| 407 | RBM25 |  |  | 8608 | -0.196 | -0.3280 | Yes |
| 408 | SPG7 |  |  | 8610 | -0.196 | -0.3237 | Yes |
| 409 | NTM |  |  | 8612 | -0.196 | -0.3193 | Yes |
| 410 | DDX17 |  |  | 8613 | -0.196 | -0.3149 | Yes |
| 411 | IGFBP3 |  |  | 8615 | -0.197 | -0.3105 | Yes |
| 412 | NOP56 |  |  | 8619 | -0.199 | -0.3063 | Yes |
| 413 | MFSD10 |  |  | 8620 | -0.199 | -0.3018 | Yes |
| 414 | CCDC47 |  |  | 8621 | -0.199 | -0.2973 | Yes |
| 415 | ZNF397 |  |  | 8623 | -0.200 | -0.2929 | Yes |
| 416 | ATP2A2 |  |  | 8624 | -0.200 | -0.2883 | Yes |
| 417 | EWSR1 |  |  | 8626 | -0.201 | -0.2839 | Yes |
| 418 | LUC7L3 |  |  | 8631 | -0.203 | -0.2797 | Yes |
| 419 | TTC14 |  |  | 8635 | -0.205 | -0.2754 | Yes |
| 420 | SREK1 |  |  | 8636 | -0.206 | -0.2707 | Yes |
| 421 | DST |  |  | 8644 | -0.211 | -0.2668 | Yes |
| 422 | SERPINH1 |  |  | 8646 | -0.212 | -0.2621 | Yes |
| 423 | PDIA3 |  |  | 8647 | -0.212 | -0.2573 | Yes |
| 424 | CALD1 |  |  | 8649 | -0.213 | -0.2526 | Yes |
| 425 | ZNF207 |  |  | 8655 | -0.215 | -0.2483 | Yes |
| 426 | PCMT1 |  |  | 8656 | -0.215 | -0.2434 | Yes |
| 427 | VCAN |  |  | 8664 | -0.219 | -0.2392 | Yes |
| 428 | KANSL1 |  |  | 8669 | -0.223 | -0.2347 | Yes |
| 429 | SLC25A37 |  |  | 8671 | -0.224 | -0.2297 | Yes |
| 430 | POR |  |  | 8682 | -0.228 | -0.2257 | Yes |
| 431 | KTN1 |  |  | 8686 | -0.231 | -0.2208 | Yes |
| 432 | ARGLU1 |  |  | 8690 | -0.234 | -0.2159 | Yes |
| 433 | CDH2 |  |  | 8695 | -0.237 | -0.2109 | Yes |
| 434 | COL6A1 |  |  | 8698 | -0.238 | -0.2058 | Yes |
| 435 | DDOST |  |  | 8706 | -0.242 | -0.2011 | Yes |
| 436 | PDIA4 |  |  | 8712 | -0.251 | -0.1960 | Yes |
| 437 | SNTG1 |  |  | 8713 | -0.251 | -0.1903 | Yes |
| 438 | B2M |  |  | 8718 | -0.258 | -0.1849 | Yes |
| 439 | LAPTM4A |  |  | 8730 | -0.267 | -0.1802 | Yes |
| 440 | FUS |  |  | 8731 | -0.268 | -0.1741 | Yes |
| 441 | CANX |  |  | 8735 | -0.270 | -0.1683 | Yes |
| 442 | PNISR |  |  | 8737 | -0.273 | -0.1622 | Yes |
| 443 | KCNQ1OT1 |  |  | 8742 | -0.281 | -0.1563 | Yes |
| 444 | APLP2 |  |  | 8755 | -0.292 | -0.1511 | Yes |
| 445 | ADAM9 |  |  | 8756 | -0.296 | -0.1444 | Yes |
| 446 | APP |  |  | 8758 | -0.300 | -0.1377 | Yes |
| 447 | VMP1 |  |  | 8760 | -0.302 | -0.1310 | Yes |
| 448 | DYNC1H1 |  |  | 8761 | -0.302 | -0.1241 | Yes |
| 449 | CD9 |  |  | 8762 | -0.303 | -0.1172 | Yes |
| 450 | NKTR |  |  | 8766 | -0.315 | -0.1104 | Yes |
| 451 | RRBP1 |  |  | 8768 | -0.320 | -0.1032 | Yes |
| 452 | CALR |  |  | 8773 | -0.330 | -0.0962 | Yes |
| 453 | NEAT1 |  |  | 8775 | -0.334 | -0.0887 | Yes |
| 454 | ITGB1 |  |  | 8777 | -0.335 | -0.0812 | Yes |
| 455 | PPIB |  |  | 8782 | -0.351 | -0.0737 | Yes |
| 456 | PTPRZ1 |  |  | 8783 | -0.360 | -0.0656 | Yes |
| 457 | HSPA5 |  |  | 8785 | -0.369 | -0.0573 | Yes |
| 458 | MEG3 |  |  | 8786 | -0.370 | -0.0489 | Yes |
| 459 | TNC |  |  | 8787 | -0.389 | -0.0400 | Yes |
| 460 | SLC1A3 |  |  | 8788 | -0.413 | -0.0307 | Yes |
| 461 | LMAN1 |  |  | 8790 | -0.441 | -0.0208 | Yes |
| 462 | PDIA6 |  |  | 8792 | -0.448 | -0.0107 | Yes |
| 463 | HSP90B1 |  |  | 8794 | -0.487 | 0.0002 | Yes |
Table: GSEA details [plain text format]

  

Fig 2: DOROSZKO\_U3180MG\_CLUSTER\_2: Random ES distribution      
 Gene set null distribution of ES for **DOROSZKO\_U3180MG\_CLUSTER\_2**
